# Dataset related to the effect of resource-based instruction on Rwandan pre-service biology teachers' academic achievement, attitude, and motivation

**DOI:** 10.1016/j.dib.2022.107939

**Published:** 2022-02-09

**Authors:** Josiane Mukagihana, Florien Nsanganwimana, Catherine M. Aurah

**Affiliations:** a African Centre of Excellence for Innovative Teaching and Learning Mathematics and Science (ACEITLMS), University of Rwanda College of Education (URCE), Kayonza, P.O BOX 55 Rwamagana, Rwanda; b University of Rwanda College of Education (URCE), Rwanda; c Masinde Muliro University of Science and Technology, Kenya

**Keywords:** Resource-based instruction, Lecture method, Animation instruction, Small laboratory activities, Time-series design, Pre-and post-test design, Pre-service biology teachers, Rwanda

## Abstract

This dataset belongs to a large doctoral research project in Biology education conducted under the African Centre of Excellence for Innovative Teaching and Learning Mathematics and Science (ACEITLMS), University of Rwanda College of Education (URCE). The data were collected from students undertaking biology education as their future teaching career [here referred to as pre-service biology teachers]. The data were collected from two higher learning institutions in Rwanda. This data article describes how we collected data and designed the study. We used valid and reliable research instruments to collect data. Thus, this dataset informs university lecturers, education policymakers of the status of academic achievement, attitude, and motivation towards learning biology using resource-based instructions such as animations and small laboratory groups. It allows researchers to reanalyze it, depending on the interest variables.

## Specifications Table

**Table:** 

Subject	Social sciences
Specific subject area	Biology education
Type of data	Table
How data were acquired	A Pre-service Biology Teachers Achievement Test (PBTAT) [see the supplementary material] was used as an achievement test, while a Biology Attitude Scale (BAS) adapted from the Russell and Hollander [1] study was used to reveal pre-service teachers' attitudes towards learning Biology. Additionally, Academic Motivation Scale for Learning Biology (AMSLB) adapted from the Aydın, Yerdelen, Yalmancı, and Göksu [2] study was used to reveal pre-service teachers' motivation towards learning Biology. The observation was also done using classroom observation protocol for undergraduate STEM (COPUS) [3] to monitor the implementation of resource-based intervention on a time-series basis.
Data format	Cleaned raw data Analyzed data
Parameters for data collection	Participants are from two different universities. One university is public, while another university is a private university. Pre-service teachers involved are pursuing biology education program and the content focused is microbiology. Instruments used were tested in the pilot stage for reliability.
Description of data collection	The data were collected from November 2020 to March 2021. We first validated our PBTAT tool and checked its reliability—using split-half equivalent forms to the same group of students—together with our used tools. We got an ethical clearance and presented it to research sites for research permission. We then explained the study's intent to participants [here referred to as pre-service biology teachers] and agreed to participate voluntarily. We administered a pre-assessment first, we taught them differently according to various resource-based instruction (RBI), and thirdly administered the same assessments as post-evaluation of the effect of RBI both in terms of achievement, attitude, and motivation.
Data source location	Data were collected from the University of Technology and Arts of Byumba (UTAB) located in Gicumbi district, Northern Province, Rwanda, and the University of Rwanda College of Education (URCE) located in Kayonza district, Eastern Province, Rwanda.
Data accessibility	The raw data are accessible and deposited on the Mendeley repository. Direct URL to data: https://data.mendeley.com/datasets/bpfb5sdmyg/1

## Value of the Data

• Assessment of resource-based instructions that are important to improve learning of biology and enhance attitudes and motivation towards learning is essential. The dataset is helpful for university lecturers to be informed on what RBIs are vital to their teaching routine.
• The dataset significantly informs university management to evaluate lecturers teaching strategies and monitor the research-based instructions that have proved a good outcome.
• The data is available for researchers to analyze among other variables, such as how RBIs improve pre-service biology teachers at public or private universities, motivate them to learn biology, and lift their attitudes towards biology courses. Researchers may explore what activities are likely occurring in a class of lecture, animation-based, or laboratory-based instruction on the side of instructor and students.

## 1 Data Description

The dataset contains five types of data collected and distributed into eight files [4]. (a) Data related to reliability testing have one file [Reliability for PBTAT tested on equivalent forms method], (b) Data related to PBTAT have two files [Achievement data for pre-service teachers at URCE, and Achievement data for pre-service teachers at UTAB], (c) Data related to BAS have two files [Attitude data for pre-service teachers at URCE, and Attitude data for pre-service teachers at UTAB], (d) Data related to AMSLB have two files [Motivation data for pre-service teachers at URCE, and Motivation data for pre-service teachers at UTAB], and (e) Data related to COPUS have one file [Classroom observation data at UTAB]. All these data are available in the Mendeley repository at https://data.mendeley.com/datasets/bpfb5sdmyg/1. The reliability for PBTAT (in CSV format) and PBTAT questionnaire (in PDF format) is also attached to this manuscript as supplementary files.

Reliability for PBTAT was tested on the split-half method where scores for odd and even item numbers were correlated among a group of 27 students. “Achievement data for pre-service teachers at URCE” file contains six sheets where the first two sheets are pre-and post-test scores for students in the control group, the second two sheets are pre-and post-test scores for students in the first experimental group taught using animation-based instruction, and the last two sheets are pre-and post-test scores for students in the second experimental group taught in small-group laboratory activities. Each sheet shows the students’ code (anonymized name) and gender and answers to the 20 PBTAT. Question 1–7 have multiple answer choices (A–D or A–E), Question 8–11 are true or false questions, while the rest of the questions (Question 12–20) are short answer type of questions. Similar to the data at UTAB (Achievement data for pre-service teachers at UTAB), data are presented in the same way except that this file contains only four sheets due to the design used (time-series). Thus, the first sheet contains pre-test scores, the second contains post-test scores during lecture method, the third contains post-test scores during animation-based instruction, and the fourth sheet contains post-test scores during small-group laboratory activities.

In the same vein, the files related to attitude (Attitude data for pre-service teachers) and motivation (Motivation data for pre-service teachers) have similar sheets at URCE (six sheets) and similar sheets at UTAB (four sheets). The Biology Attitude Scale (BAS) adapted from the Russell and Hollander [1] comprises 14 items. In contrast, Academic Motivation Scale for Learning Biology (AMSLB) adapted from the Aydın et al. [2] comprises 19 items or statements divided into four factors (intrinsic motivation [item 1–6], motivation [item 7–11], extrinsic motivation - career [item 12–15], and extrinsic motivation - social [item 16–19]). Differently, Classroom observation data were collected only at UTAB and contains four sheets. The first three sheets present respectively the data for lecture, animation, and lab class while the fourth shows the key observation codes meaning as explained by the COPUS developers [3]. The filled cells (by ones) show where a certain teaching and learning activity was realized, while empty cells show nothing observed.

Table 1 shows the characteristics and number of participants in each study site. Thirty pre-service biology teachers participated both before and after intervention and performed achievement tests at UTAB. Thirty-four pre-service biology teachers completed the attitude scale, while 33 completed the motivation questionnaire. Likewise, 174 pre-service biology teachers completed the achievement test, 171 completed the attitude scale, while 179 completed the motivation scale at URCE.

**Table 1:** Number of participants along with RBI intervention groups.

UTAB			
Intervention groups	Achievement test	Attitude scale	Motivation questionnaire
Pre-assessment	30	34	33
Post-assessment	30	34	33
Total	30	34	33


URCE			
Intervention groups	Achievement test	Attitude test	Motivation test
Lecture	60	59	60
Animation	55	60	59
Laboratory	59	52	60
Total	174	171	179

## 2 Experimental Design, Materials and Methods

We collected data from two different university or higher learning institutions in Rwanda between November 2020 and March 2021. One is the University of Rwanda College of Education (URCE) located in Kayonza district, Eastern province, while another is the University of Technology and Arts of Byumba (UTAB) located in Gicumbi district, Northern province. We implemented a quasi-experimental of the non-equivalent group, pre-test, and post-test control group design [5] at URCE and Equivalent time-series design [6] at UTAB due to limited sample. We used four research tools to check the effect of various resource-based instructions (RBIs). (a) The Pre-service Biology Teachers Achievement Test (PBTAT) was developed by the researchers and validated by lecturers at URCE. The test is available in the supplementary material attached to this text. Its reliability data is available in one file uploaded in the Mendeley repository as described in the data description section. (b) We used the Biology Attitude Scale (BAS) of Russell and Hollander [1] to test how pre-service teachers increase their attitudes towards learning Biology. (c) We used the Academic Motivation Scale for Learning Biology (AMSLB) of Aydın et al. [2] to test which RBIs motivate pre-service teachers' to learn Biology. (d) We observed class during implementing RBIs on time-series design at UTAB using the classroom observation protocol for undergraduate STEM (COPUS) of Smith et al. [3], and three lessons were observed during lecture method, five were observed during animation-based instruction. In contrast, four were observed during small groups of laboratory activities. Detailed information on collecting and analyzing data is found in Ndihokubwayo et al. [7] study.

## Ethics Statement

We first applied for ethical clearance from the research and innovation unit at the University of Rwanda College of Education (URCE). An ethical clearance [Ref: 01/P-CE/568/EN/gi/2019] was provided after evaluating our proposal and research instruments. We sought permission from UTAB and URCE departments to conduct the research with their students and allow the first author to interact with the students as an instructor. Students have been explained the purpose of the study and issued full voluntary participation and rights to withdraw at any time.

## CRediT authorship contribution statement

**Josiane Mukagihana:** Conceptualization, Methodology, Data curation, Formal analysis, Writing – original draft. **Florien Nsanganwimana:** Conceptualization, Validation, Writing – review & editing, Supervision. **Catherine M. Aurah:** Conceptualization, Visualization, Writing – review & editing, Supervision.

## Declaration of Competing Interest

The authors declare that they have no known competing financial interests or personal relationships which have or could be perceived to have influenced the work reported in this article.

## Appendix Supplementary Materials

Image, application 1

Image, application 2
