# Supplementary material for: Dataset related to the effect of resource-based instruction on Rwandan pre-service biology teachers' academic achievement, attitude, and motivation
Source: Data Brief. 2022 Feb 9;41:107939. doi: 10.1016/j.dib.2022.107939 (PMC8857598; doi:10.1016/j.dib.2022.107939)
Supplement: Supplementary file 1 [file mmc1.pdf]

COLLEGE OF EDUCATION

African Center of Excellence for Innovative Teaching and Learning Mathematics and Science (ACEITLMS)

Pre-service Biology Teachers Achievement Test: Module of microbiology.

Description:

Dear pre-service biology teachers, the questions bellow were prepared for measuring your academic achievements generally in biology considering your performance in module of microbiology. The types of questions in the test are objective questions (multiple and true or false) and short answer questions.

IDENTIFICATION

University name:

Reg number:

Gender:

INSTRUCTIONS:

- Answer all questions. -Read carefully each question and provide the correct answer.

Date: ……………………. Duration: **40min**

Multiple choice questions.

1. Which of the following is a reason that microorganisms are useful in many different research laboratories?
   A. They are easy to see and count
   B. They have fairly complex structures and are expensive
   C. They reproduce quickly and grow in large numbers
   D. They live everywhere so contaminants from the environment are not a problem

2. Prokaryotes do not have which of the following?
   A**.** Cell membrane
   B**.** Nucleus' membrane
   C**.** Cytoplasm
   D**.** Ribosomes
3. The Common basic stains are
   A .methylene blue
   B. crystal violet,
   C. safranin
   D. malachite green.
   E. all of the above.

4. Which of the following correlate with exponential phase?
   A. Log phase
   B. Rate of growth is constant and also called as balanced growth
   C. Population is most uniform in terms of chemical and physical properties during this phase.
   D. Exponential growth- all cellular components are synthesized at a constant rate.
   E. All of the above

5. Gram-negative bacteria have much of …………..and that is found on their outer membrane
   A. Lipopolysaccharides
   B. Peptidoglycan
   C. Teichoic acid
   D. All the above

6. Among the following method of microbial culture, what is the most common and suitable for isolation of pure culture?

   A. Pour plate method

   B. Streak Plate method

   C. Dilution method

   D. None of the above

7. Among the following types of stains what is used to identify bacteria and that was developed by a Danish physician Hans Christian Gram.

   A. Simple staining

   B. Differential staining

   C. Gram stain
   D. All The above

<u>True or False questions.</u>

8. **A *stain*** is a chemical that adheres to structures of the microorganism as dyes so that microorganism can be easily seen under a microscope. **True or False?**

9. *Staphylococcus* and *Streptococcus* are examples of Gram-positive bacteria. **True or False?**

10. Agar is a complex polysaccharide used as solidifying agent for culture media preparation. **True or False?**

11. **A.** Bacteria generation time is simply the time it takes for one cell to divide into two
True or False?

<u>Short answer questions</u>

12. The concept that human and animal diseases are caused by microorganisms is called…………
13. Gram positive bacteria stain in ……………. color

14. A peptidoglycan layer that is very thick is commonly seen in.............................Bacteria.

15. Lawn culture is used for different purpose including bacteria antibiotic sensitivity testing?

Yes or No

16. Who discovered the fungus Penicillium that produced an antibiotic called penicillin in 1929?

17. Who laid the foundation of aseptic techniques that prevent contamination by unwanted microbes?

18. Who was the first person to use a microscope to observe living cells?

19. Microbial cultures are used to determine the type of organism, its abundance in the sample being tested? **Yes Or NO**

20. Aside from peptidoglycan, what other component makes up a large percentage of the gram positive cell wall?
